# Renal Recovery After Acute Kidney Injury and Long-term Outcomes: Is Time of the Essence?

**DOI:** 10.1001/jamanetworkopen.2020.2676

**Published:** 2020-04-01

**Authors:** Ravindra L. Mehta

**Affiliations:** Division of Nephrology, University of California, San Diego, La Jolla.

After any illness, restoration to good health results from organ functional recovery that is dependent on the prior state of health, severity of illness, and the process of care. Although the severity stage can be delineated by the magnitude of structural and functional derangement at any point in time, quantifying recovery is often difficult, as it requires knowledge of the prior condition of the organ and ongoing interventions. The course of disease, with progression or improvement, consequently represents both the nature and extent of injury and repair, the associated comorbidities, and the management. Furthermore, the association of the extent of recovery with short-term and long-term outcomes requires accurate definitions and recognition of the events during and after the illness. In acute kidney injury (AKI), it is well recognized that the stage of AKI; associated comorbidities of diabetes, heart failure, and chronic kidney disease (CKD); and dialysis requirements are associated with adverse outcomes.^1^ More recently, attention has focused on the duration and number of episodes of AKI as factors associated with nonrecovery from AKI and short-term and long-term mortality. Most of these data have emerged from retrospective studies from postcardiac surgery and intensive care unit settings and have not been studied prospectively in other settings. Bhatraju et al^2^ studied long-term major adverse kidney events (MAKE), including incidence or progression of CKD, death, or dialysis, in the prospective, multicenter Assessment, Serial Evaluation, and Subsequent Sequelae (ASSESS-AKI) cohort study. Patients with and without AKI who survived hospitalization for 3 months were enrolled and followed up for a median of 4.7 years. Patients with AKI were classified as having resolving AKI if their serum creatinine values decreased by 0.3 mg/dL or more (to convert to micromoles per liter, multiply by 88.4) or 25% or more from maximum within the first 72 hours after AKI diagnosis and as having nonresolving AKI if they failed to meet these criteria. Most patients (74%) had Stage 1 AKI, 9% had sepsis, 4% required mechanical ventilation, and less than 7% needed dialysis. Of the patients with AKI, 62% had a resolving pattern; 54% of these patients had returned to baseline creatinine concentration at hospital discharge and 51% returned to baseline creatinine concentration at 3 months, whereas only 16% of patients with nonresolving AKI had returned to baseline creatinine concentration at hospital discharge and 38% returned to baseline creatinine concentration at 3 months. Compared with patients with no AKI, those with resolving AKI had an almost 2-fold higher risk of MAKE and those with nonresolving AKI had an almost 3-fold higher risk of MAKE; these risks persisted when adjusted for underlying comorbidities and Kidney Disease: Improving Global Outcomes stage of AKI. Patients with nonresolving AKI had a higher rate of incident and progressive CKD. Patients with AKI had a higher mortality rate than those without AKI (22% vs 12%), but there were no differences between patients with resolving AKI and those with nonresolving AKI. Interestingly, the AKI severity stage did not differentiate the development of MAKE during the 4 years of follow-up.

The findings in the study by Bhatraju et al^2^ provide evidence for considering the timing of functional recovery from AKI as a factor associated with future adverse events. Prior studies in different settings have examined these issues. In a cohort of 10 275 patients undergoing coronary artery bypass grafting, Swaminathan et al^3^ showed the percentage decline in serum creatinine concentration within the first 24 hours from the peak value was the factor best associated with 1-year survival in the 1113 patients with AKI. Kellum et al^4^ determined 5 different patterns of renal recovery after AKI in a single-center cohort of patients in the intensive care unit and showed that those with sustained nonrecovery had the highest mortality during hospitalization. Most recently, Siew et al^5^ analyzed a retrospective cohort of 47 903 predominantly male US veterans with Kidney Disease: Improving Global Outcomes Stage 2 and 3 AKI and stratified their recovery pattern based on the time to achieve a serum creatinine concentration within 120% of a baseline value within 90 days of the peak injury. During a median of 42 months of follow-up, patients with recovery within 1 to 4 days had the lowest rates of kidney failure or a continued 40% decrease in estimated glomerular filtreation rate below a reference value during the 90-day recovery period. The study by Bhatraju et al^2^ study additionally highlights the shortfall of current AKI staging criteria used alone in assessing outcomes, as the severity stage by itself did not differentiate among which patients would develop MAKE; rather, the recovery pattern was more informative. This finding was similarly seen in the coronary artery bypass graft^3^ and intensive care unit^4^ cohort studies and in aggregate emphasizes the need for clinicians to consider the patient’s course after AKI as a determinant of outcome.

Although there is now increasing interest in understanding the timing of recovery to identify distinct phenotypes prone to adverse outcomes, it is important to realize that these are just initial pieces of the puzzle. For instance, the ability to distinguish recovery from nonrecovery and the timing thereof depends on the definitions used. Xu et al^6^ compared 5 prevailing definitions of kidney recovery in a cohort of patients who underwent cardiac surgery and had AKI and found recovery rates from 44% to 84%, illustrating the need for uniform definitions of kidney recovery. Factors associated with the distinction between an early vs delayed recovery are also subject to variation, as the time to recovery is established from the peak value of serum creatinine. Although peak values are determined by the underlying severity of AKI, they are also associated with the process of care applied. Few studies describe a creatinine value corrected for cumulative fluid accumulation; hence, the ascertainment of peak values and improvement may not be accurate. Comorbidities including underlying CKD are recognized as risk factors for AKI and are associated with renal recovery. In the study by Bhatraju et al^2^, 40% of the patients had underlying CKD; however, incident CKD was the major MAKE component that differed between the resolving AKI and nonresolving AKI groups and the rate of CKD progression was not significantly different between groups. The higher risk for MAKE among patients with nonresolving AKI was sustained when patients with CKD were excluded. It is likely that intervening events (eg, repeated AKI episodes or continued nephrotoxic exposures) are associated with the patients’ course after an AKI episode and may not be captured in the study population. The ASSESS-AKI study design limited enrollment to patients who survived 90 days; hence, most of the patients had mild AKI and a very low need for dialysis. The long intervals between study assessments could limit recognition of prevailing events that are associated with the course of illness and adverse outcomes.

The cumulative evidence to date highlights the need for further research in this area and could be guided by a framework to understand the association among patient susceptibility, exposures, process of care, and outcomes.^1^ The availability of big data, machine learning and algorithms for data mining and biomarkers offers novel opportunities to improve our understanding of these complex interactions.^7,8^ It is, however, clear that clinicians managing patients with AKI should consider the severity of the disease and the ensuing course and tailor their diagnostic and therapeutic interventions to facilitate rapid and complete recovery of kidney function. Ultimately, restoring good health is the goal for which both patients and physicians are striving.
